# Dawn-to-dusk dry fasting decreases circulating inflammatory cytokines in subjects with increased body mass index

**DOI:** 10.1016/j.metop.2024.100274

**Published:** 2024-02-07

**Authors:** Zahraa Al lami, Miray Kurtca, Moin Uddin Atique, Antone R. Opekun, Mohamad S. Siam, Prasun K. Jalal, Bijan Najafi, Sridevi Devaraj, Ayse L. Mindikoglu

**Affiliations:** a Margaret M. and Albert B. Alkek Department of Medicine, Section of Gastroenterology and Hepatology, Baylor College of Medicine, Houston, TX, USA; b Clinical Chemistry and Point of Care Technology, Texas Children's Hospital and Health Centers, Department of Pathology and Immunology, Baylor College of Medicine, Houston, TX, USA; c Interdisciplinary Consortium on Advanced Motion Performance (iCAMP), Michael E. DeBakey Department of Surgery, Baylor College of Medicine, Houston, TX, USA; d Department of Pediatrics, Division of Gastroenterology, Nutrition and Hepatology, Baylor College of Medicine, Houston, TX, USA; e Michael E. DeBakey Department of Surgery, Division of Abdominal Transplantation, Baylor College of Medicine, Houston, TX, USA

**Keywords:** Dry fasting, cytokine, Inflammation, Intermittent fasting, Dawn-to-dusk dry fasting, Diurnal fasting, Daytime fasting, Obesity

## Abstract

**Background:**

The circadian rhythm involves numerous metabolic processes, including sleep/awakening, body temperature regulation, hormone secretion, hepatic function, cellular plasticity, and cytokine release (inflammation), that appear to have a dynamic relationship with all the processes above. Studies have linked various cytokines to the chronic state of low-grade inflammation and oxidative stress in obesity. Dawn-to-dusk dry fasting (DDDF) could alleviate the adverse effects of obesity by decreasing inflammation. This study examined the effects of DDDF on circulating inflammatory cytokines in subjects with increased body mass index (BMI).

**Methods:**

The current observational prospective study included adult subjects with a BMI equal to or greater than 25 kg/m^2^ who practiced the annual religious 30-day DDDF. Individuals with significant underlying medical conditions were excluded to limit confounding factors. All subjects were evaluated within two weeks before 30-day DDDF, within the fourth week of 30-day DDDF, and within two weeks after 30-day DDDF. Multiple cytokines and clinical health indicators were measured at each evaluation.

**Results:**

Thirteen subjects (10 men and three women) with a mean age of 32.9 years (SD = 9.7 years) and a mean BMI of 32 kg/m2 (SD = 4.6 kg/m^2^) were included. An overall associated decrease in the levels of multiple cytokines with DDDF was observed. A significant decrease in the mean interleukin 1 beta level was observed within the fourth week of 30-day DDDF (P = 0.045), which persisted even after the fasting period (P = 0.024). There was also a significant decrease in the mean levels of interleukin 15 (IL-15) (P = 0.014), interleukin 1 receptor antagonist (P = 0.041), macrophage-derived chemokine (MDC) (P = 0.013), and monokine induced by interferon gamma/chemokine (C-X-C motif) ligand 9 (P = 0.027) within the fourth week of 30-day DDDF and in the mean levels of fibroblast growth factor 2 (P = 0.010), interleukin 12 p40 subunit (P = 0.038), interleukin 22 (P = 0.025) and tumor necrosis factor alpha (P = 0.046) within two weeks after 30-DDDF. In terms of anthropometric parameters, there was a decrease in mean body weight (P = 0.032), BMI (P = 0.028), and hip circumference (P = 0.007) within the fourth week of 30-day DDDF and a decrease in mean weight (P = 0.026), BMI (P = 0.033) and hip circumference (P = 0.016) within two weeks after 30-day DDDF compared with the levels measured within two weeks before 30-day DDDF. Although there was no significant correlation between changes in weight and changes in circulating inflammatory cytokines, there was a significant positive correlation between changes in waist circumference and changes in specific inflammatory cytokines (e.g., IL-15, MDC, platelet-derived growth factor, soluble CD40L, vascular endothelial growth factor A) within the fourth week of 30-day DDDF and/or two weeks after 30-day DDDF. A significant decrease in mean average resting heart rate within the fourth week of 30-day DDDF was also observed (P = 0.023), and changes between average resting heart rate and changes in interleukin-8 levels within the fourth week of 30-day DDDF compared with baseline levels were positively correlated (r = 0.57, P = 0.042).

**Conclusion:**

DDDF appears to be a unique and potent treatment to reduce low-grade chronic inflammation caused by obesity and visceral adiposity. Further studies with more extended follow-up periods are warranted to investigate the long-term anti-inflammatory benefits of DDDF in individuals with increased BMI.

## 1 Introduction

The obesity epidemic is a significant challenge to the healthcare system [1]. Obesity plays a role in multiple chronic diseases, including type II diabetes mellitus, metabolic syndrome, atherosclerotic cardiovascular disease, metabolic dysfunction-associated steatotic liver disease (MASLD), MASLD-induced cirrhosis, chronic kidney disease, malignancies, and even mental health problems and early aging [[2], [3], [4], [5]]. Numerous initiatives have been implemented worldwide in an attempt to reverse the continuous increase in the average body weight without significant success [6]. Over one-third of the world's adult population is overweight and at risk for accelerated aging and early death due to obesity-associated co-morbidities [5,7].

Obesity is a poorly understood, complex, and heterogeneous disorder that undoubtedly includes genetic predisposition, epigenetic, and environmental factors that are challenging to treat and to prevent. Obesity appears to develop secondary to energy imbalance [8,9], but the driving forces behind obesity remain unclear. In some cases, when calories are consumed in excess to meet the needs of energy homeostasis, conservation occurs, which appears to predispose to obesity and its consequences. It is unclear if obesity occurs because of metabolic maladaptation, perhaps as a by-product of positive selection and/or colonic dysbiosis [10]. Beyond this, the “body weight set-point” appears to be a moving target in the positive direction, and the factors responsible for this epiphenomenon are unknown [11].

Caloric intake exceeding total body energy expenditure has been thought to result in increased accumulation of fat molecules within the adipose tissue with an inflammatory response associated with adipocyte hypertrophy and hyperplasia [12]. Low-grade chronic systemic inflammation may contribute to a disruption of energy homeostasis, and resultant obesity occurs for reasons that transcend the passive accumulation of excess body weight in time of abundance [8,[12], [13], [14]]. Adipocyte hypertrophy also triggers regional hypoxia, resulting in the secretion of inflammatory cytokines, and creates a constant state of chronic systemic inflammation [[12], [13], [14], [15]].

Other investigators and we previously reported that dawn-to-dusk dry fasting (DDDF) alleviates inflammation and oxidative stress in obese and non-obese individuals [[16], [17], [18], [19], [20], [21]]. It has been suggested that the anti-inflammatory benefits of DDDF could be due to a decrease in the overall size of the visceral adipose tissue, a known source of proinflammatory markers, and downregulation of the inflammatory and oxidative pathways gene expression [[16], [17], [18], [19], [20], [21]]. Several existing intermittent fasting or time-restricted eating (TRE) regimens allow water drinking and/or non-caloric drinks during the fasting window and appear counter to DDDF. DDDF appears to be a superior alternative to existing water and wet fasting regimens to achieve robust anti-inflammatory effects: (1) Dawn and dusk are critical periods of the 24-h circadian cycle in human metabolism [22,23]. Subjects who perform DDDF have their meals consistently at dawn and dusk, and this would align the phase of peripheral circadian clocks with the phase of central clock entrained to local dawn and dusk, ensuring circadian rhythm compliance [19]. Meal timing in DDDF contrasts meal timing in several TRE regimens. In many reported TRE regimens, drinking at random times, skipping breakfast, and having late-night eating likely disrupt circadian regulation of metabolism and energy balance by misaligning peripheral clocks with the central clock entrained to dawn and dusk [[24], [25], [26], [27], [28], [29]]; 2) Dry fasting prevents protein degradation as an alternative energy source during fasting and therefore has a protective effect against skeletal muscle and vital body weight loss that can occur with prolonged water/wet fasting [30]. A TRE regimen that allowed water and non-caloric drinks during fasting resulted in a significant lean muscle loss [29], and this could be secondary to inhibition of vasopressin due to drinking and then in turn, the inhibition of epinephrine resulting in protein breakdown [[30], [31], [32], [33], [34]]. Epinephrine depletion has been shown to increase protein breakdown during fasting and this is likely caused by the inactivation of lipoprotein lipase, which prevents fatty acids from being used as an alternative energy source to glucose [[30], [31], [32], [33], [34], [35], [36]]; 3) Contrary to dehydration that can occur with prolonged dry fasting regimens, DDDF was shown to be safe in various populations [[37], [38], [39], [40], [41], [42], [43]]. Potential dehydration can be easily prevented if adequate hydration is achieved during the non-fasting period of DDDF and necessary health precautions are taken [44]. A recent observational study of 34 subjects who practiced DDDF showed that the plasma osmolality and 24-h urine osmolality were within the normal physiological range during the entire daylight dry fasting period [41]; 4) Shorter dry fasting cycles from dawn-to-dusk prevent the risk of refeeding syndrome [45], overfeeding and weight gain that can occur with prolonged fasting regimens.

Previous studies reported the effect of dry fasting on a limited number of inflammatory cytokines/inflammatory biomarkers [17,20,21,46]. Our earlier study showed that 4-week DDDF induced an anti-inflammatory and anti-tumorigenic proteome in the peripheral blood mononuclear cells in 14 subjects with metabolic syndrome, twelve of whom with MASLD [19]. In this study, we aimed to evaluate the anti-inflammatory effect of DDDF further. For this, we determined the effect of 30-day DDDF on circulating inflammatory cytokines/inflammatory biomarkers along with weight, body mass index, surrogate markers of visceral adiposity, including waist circumference and waist-to-hip ratio (WHR) [47], and other health and fitness parameters in subjects with a body mass index (BMI) within the World Health Organization BMI categories of overweight and obesity (defined as BMI equal to or greater than 25 kg/m2) [7].

## 2 Methods

### 2.1 Study subjects

The study approval was obtained from the Institutional Review Board for Human Subject Research for Baylor College of Medicine and Affiliated Hospitals (BCM IRB) (Protocol number H-31612). Subjects who were going to observe the religious practice of DDDF (without eating or drinking from dawn to dusk) [48] were included in the study. Subjects were included if they were 18 years old or older and had a BMI equal to or greater than 25 kg/m^2^. Potential subjects were excluded if they had any of the following: 1) plans for international travel during the study period; 2) plans for night shift work during the study period; 3) inability to provide informed consent; 4) pregnancy or breastfeeding; 5) active cancer; 6) active infection requiring antibiotic treatment; 7) seizure disorder; 8) cardiovascular event during the last six months before the enrollment; 9) and the use of alcohol or recreational substances.

### 2.2 Study procedures

#### 2.2.1 Health parameters measured at study visits

Subjects were scheduled for three visits at Baylor College of Medicine in the Texas Medical Center Digestive Diseases Center Clinical Research Core E Laboratory, including Visit 1 within two weeks before 30-day DDDF, Visit 2 within the fourth week of the 30-day DDDF, and Visit 3 within two weeks after 30-day DDDF. During Visit 1, written informed consent was obtained, subject eligibility was determined, and medical history and physical examination were performed. A urine pregnancy test was performed on female subjects of childbearing age. In each study visit, temperature was checked, anthropometric measurements including weight, height, waist, and hip circumference were performed, resting systolic and diastolic blood pressure and heart rate were measured, and fasting (minimum 8 h after the last food and drink intake) blood samples were collected and timely processed by the investigators at the point of care to minimize deterioration.

#### 2.2.2 Circulating inflammatory cytokines/inflammatory biomarkers

Circulating cytokines were measured within two weeks before the 30-day DDDF (Visit 1), within the fourth week of the 30-day DDDF (Visit 2), and within two weeks after 30-day DDDF (Visit 3). Fibroblast growth factor 2 (FGF-2), fms-related receptor tyrosine kinase 3 ligand (FLT3L), granulocyte colony-stimulating factor (GCSF), interferon gamma (IFN-gamma), interleukin 10 (IL-10), interleukin 12 p40 subunit (IL-12p40), interleukin 13 (IL-13), interleukin 15 (IL-15), interleukin 1 beta (IL-1 beta), interleukin 1 receptor antagonist (IL-1ra), interleukin 22 (IL-22), interleukin 6 (IL-6), interleukin 8 (IL-8), interferon gamma inducible protein 10 (IP-10), macrophage-derived chemokine (MDC), monokine induced by interferon gamma (MIG)/chemokine (C-X-C motif) ligand 9 (CXCL9), platelet-derived growth factor (PDGF-AB/BB), soluble CD40L (sCD40L), tumor necrosis factor alpha (TNF-alpha), and vascular endothelial growth factor A (VEGF-A) were measured in the plasma or serum of the same individuals from Visits 1, 2 and 3 using Luminex®xMAP® technology based on multiplex bead-based immunoassay. Millipore HCYTOMAG-60K-MILLIPLEX® MAP Human Cytokine/Chemokine Magnetic Bead Panel - Immunology Multiplex Assay kit was used for measurement. C-reactive protein (CRP) was measured by ELISA from ALPCO®. Cytokine and inflammatory biomarker levels below or above the detection limit were set to the limit of detection value.

#### 2.2.3 Health and fitness parameters measured by smartwatch

During Visit 1, each subject received a Garmin Vivosmart® 4 fitness tracker to monitor health and fitness parameters [49]. Subjects were instructed to wear this smartwatch for at least three days within two weeks before 30-day DDDF, within the fourth week of the 30-day DDDF, and within two weeks after 30-day DDDF. The health and fitness parameters measured by the smartwatch included heart rate, body battery (measures daily energy reserves), intensity minutes, movement (total steps, distance, floors climbed up and down), stress levels (estimated by Firstbeat Analytics engine [50]), calories burned, sleep stages and sleep pulse oximetry.

#### 2.2.4 Statistical analysis

Statistical analysis was performed using SAS software, Version 9.4 TS Level 1M7 X64_10PRO platform (SAS Institute Inc., Cary, NC, USA) [51]. A two-tailed P value of < 0.05 was considered significant for all analyses.

Paired t-tests were used to detect statistically significant differences in the variable levels (anthropometric parameters, systolic and diastolic blood pressure, mean arterial blood pressure, heart rate, inflammatory cytokines/inflammatory biomarkers, and health and fitness parameters measured by smartwatch) within the fourth week of 30-day DDDF and within two weeks after 30-day DDDF compared with the levels measured within two weeks before 30-day DDDF.

Pearson's correlation coefficients were calculated to determine statistically significant correlations between changes in the levels of inflammatory cytokines/inflammatory biomarkers and changes in the levels of anthropometric parameters, systolic and diastolic blood pressure, mean arterial blood pressure, heart rate, and health and fitness parameters measured by smartwatch within the fourth week of 30-day DDDF, and within two weeks after 30-day DDDF compared with the levels measured within two weeks before 30-day DDDF.

## 3 Results

### 3.1 Subjects

Thirteen subjects (10 men and three women), with a mean age of 32.9 years [SD = 9.7 years]) were enrolled in the study. All subjects fasted from dawn to dusk for more than 13 h daily for 30 days, from April 02, 2022, until May 01, 2022 (included). The minimum required duration of daily DDDF was 13 h, 9 min for the shortest day (April 02, 2022) and 14 h, 1 min for the longest day of the religious month (May 01, 2022). All subjects tolerated 30-day DDDF well without any reported adverse events or complications.

The first blood collection occurred within two weeks before 30-day DDDF (Visit 1) on average 8.0 days (SD = 2.8 days) before the initiation of 30-day DDDF, the second blood collection occurred within the fourth week of 30-day DDDF (Visit 2) on average after 24.9 days (SD = 1.8 days) after the initiation of 30-day DDDF, and the third blood collection occurred within two weeks after the end of 30-day DDDF (Visit 3) on average 10.2 days (SD = 1.4 days) after the completion of 30-day DDDF.

#### 3.1.1 Anthropometric parameters, blood pressure and heart rate measured at study visits

Table 1 shows the mean levels of anthropometric parameters, systolic, diastolic, and mean arterial blood pressure, and heart rate within two weeks before 30-day DDDF and their mean paired changes within the fourth week of 30-day DDDF and within two weeks after 30-day DDDF compared with baseline. There was a significant decrease in weight (P = 0.032), BMI (P = 0.028), and hip circumference (P = 0.007) within the fourth week of 30-day DDDF and a significant decrease in weight (P = 0.026), BMI (P = 0.033) and hip circumference (P = 0.016) within two weeks after 30-day DDDF compared with the levels measured within two weeks before initiation of 30-day DDDF.

**Table 1:** The effect of 30-day dawn-to-dusk dry fasting (DDDF) on anthropometric parameters, blood pressure, and heart rate in subjects with body mass index ≥25 kg/m^2^.

Parameters	The levels measured within two weeks before 30-day DDDF		The levels measured within the fourth week of 30-day DDDF compared with the levels measured within two weeks before 30-day DDDF			The levels measured within two weeks after 30-day DDDF compared with the levels measured within two weeks before 30-day DDDF		
	Mean	SD	Mean paired change^a^	SD	Paired P value	Mean paired change^a^	SD	Paired P value
Weight (kg)	94.1	14.0	−1.3	2.0	0.032	−1.5	2.2	0.026
Body mass index (kg/m^2^)	32.0	4.6	−0.4	0.6	0.028	−0.5	0.7	0.033
Waist circumference (cm)	101.8	12.0	0.1	4.6	0.927	0.04	4.3	0.975
Hip circumference (cm)	115.8	7.8	−1.4	1.3	0.007	−1.6	1.9	0.016
Waist-to-hip ratio	0.9	0.1	−0.0003	0.04	0.981	0.008	0.04	0.489
Systolic blood pressure (mmHg)	129.8	13.6	−6.0	12.2	0.102	−5.6	14.2	0.180
Diastolic blood pressure (mmHg)	80.3	8.5	−2.2	7.3	0.290	−3.5	8.4	0.154
Mean arterial blood pressure (mmHg)	96.8	9.0	−3.5	7.0	0.097	−4.2	7.9	0.076
Heart rate (beats/min)	78.7	13.7	−1.4	13.7	0.723	−2.0	12.9	0.587

a Positive and negative mean paired changes indicate an increase and a decrease, respectively, in the levels measured within the fourth week of 30-day DDDF and within two weeks after 30-day DDDF compared with the levels measured within two weeks before 30-day DDDF. SD = standard deviation.

### 3.2 Circulating inflammatory cytokines/inflammatory biomarkers

**Fig. 1:**
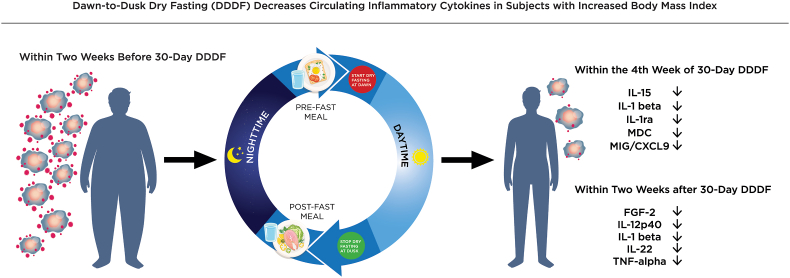
There was a significant decrease in the mean levels of multiple circulating inflammatory cytokines within the fourth week of 30-day DDDF and/or within two weeks after 30-day DDDF compared with the levels within two weeks before the initiation of 30-DDDF. There was also a significant decrease in mean body weight (P = 0.032), BMI (P = 0.028), and hip circumference (P = 0.007) within the fourth week of 30-day DDDF, and a significant decrease in mean body weight (P = 0.026), BMI (P = 0.033) and hip circumference (P = 0.016) within two weeks after 30-day DDDF compared with the levels within two weeks before of 30-day DDDF.

Table 2 shows the mean serum cytokine/inflammatory biomarker levels within two weeks before 30-day DDDF and their mean paired changes within the fourth week of 30-day DDRF and within two weeks after 30-day DDDF compared with baseline. Overall, there was a reduction in the mean levels of multiple cytokines (Fig. 1, Table 2). The subjects experienced a statistically significant reduction in the mean level of IL-1 beta within the fourth week of 30-day DDDF (P = 0.045), which persisted even after the fasting period (P = 0.024). There was a significant reduction in the mean level of IL-15 (P = 0.014), IL-1ra (P = 0.041), MDC (P = 0.013), and MIG/CXCL9 (P = 0.027) within the fourth week of 30-day DDDF compared with the baseline levels of these cytokines. There was a notable decreasing trend in the mean levels of FGF-2, IL-12p40, IL-22, and TNF-alpha within the fourth week of 30-DDDF and within two weeks after 30-DDDF, which was initially not statistically significant within the fourth week of 30-day DDDF; however, a statistically significant decrease was observed within two weeks after 30-day DDDF (P = 0.010 for FGF-2, P = 0.038 for IL-12p40, P = 0.025 for IL-22, and P = 0.046 for TNF-alpha).

**Table 2:** The effect of 30-day dawn-to-dusk dry fasting (DDDF) on circulating inflammatory cytokine/inflammatory biomarker levels in subjects with body mass index ≥25 kg/m^2^.

Cytokines	The levels measured within two weeks before 30-day DDDF		The levels measured within the fourth week of 30-day DDDF compared with the levels measured within two weeks before 30-day DDDF			The levels measured within two weeks after 30-day DDDF compared with the levels measured within two weeks before 30-day DDDF		
	Mean	SD	Mean paired change^a^	SD	Paired P value	Mean paired change^a^	SD	Paired P value
FGF-2 (pg/ml)	61.6	26.5	−7.9	15.7	0.096	−10.3	12.1	0.010
FLT3L (pg/ml)	6.3	4.2	−1.2	2.6	0.128	−0.1	4.0	0.939
GCSF (pg/ml)	57.5	26.2	−3.1	29.0	0.707	−6.3	23.1	0.343
IFN-gamma (pg/ml)	3.3	4.1	−1.4	3.6	0.202	−1.2	3.6	0.242
IL-10 (pg/ml)	13.5	10.6	−0.5	8.0	0.812	0.2	9.6	0.935
IL-12p40 (pg/ml)	80.2	56.2	−5.1	21.6	0.410	−18.7	29.0	0.038
IL-13 (pg/ml)	12.6	7.3	−1.8	6.7	0.345	−1.3	9.8	0.634
IL-15 (pg/ml)	5.0	2.2	−1.0	1.2	0.014	−0.6	2.0	0.269
IL-1 beta (pg/ml)	6.1	4.4	−1.3	2.2	0.045	−1.5	2.1	0.024
IL-1ra (pg/ml)	52.4	36.0	−9.4	14.8	0.041	−10.3	22.8	0.131
IL-22 (pg/ml)	28.4	16.8	−5.5	11.8	0.120	−5.6	7.8	0.025
IL-6 (pg/ml)	3.9	5.0	−1.0	5.6	0.526	−1.5	5.1	0.297
IL-8 (pg/ml)	3.7	3.3	−1.2	3.7	0.273	−1.4	4.2	0.247
IP-10 (pg/ml)	129.2	96.5	−34.4	84.0	0.165	−18.9	92.9	0.477
MDC (pg/ml)	308.8	80.7	−49.9	62.2	0.013	48.6	128.8	0.199
MIG/CXCL9 (pg/ml)	2199.3	1178.1	−504.1	720.9	0.027	−288.8	679.5	0.151
PDGF-AB/BB (pg/ml)	5967.7	10636.3	−2989.6	11107.4	0.351	575.5	17933.1	0.910
sCD40L (pg/ml)	1054.6	1857.1	−278.4	2054.8	0.634	934.0	5678.3	0.564
TNF-alpha (pg/ml)	22.5	14.2	−2.8	6.8	0.169	−3.3	5.3	0.046
VEGF-A (pg/ml)	26.6	66.7	−17.9	69.4	0.370	8.0	133.4	0.833
CRP (ng/ml)	7334.2	8783.7	−505.7	2927.2	0.545	−304.3	2013.1	0.596

a Positive and negative mean paired changes indicate an increase and a decrease, respectively, in the levels measured within the fourth week of 30-day DDDF and within two weeks after 30-day DDDF compared with the levels measured within two weeks before 30-day DDDF. SD = standard deviation.

### 3.3 Health and fitness parameters measured by smartwatch

Table 3 outlines the mean health and fitness parameters levels measured by smartwatch within two weeks before 30-day DDDF and their mean paired changes within the fourth week of 30-day DDRF and within two weeks after 30-day DDDF compared with baseline. There was a significant decrease in the mean average resting heart rate within the fourth week of 30-day DDDF (−2 bpm, P = 0.023). There was a statistically significant decrease in the mean level of total sleep time, light sleep time, and rapid eye movement (REM) sleep time within the fourth week of 30-day DDDF (P = 0.008 for total sleep time, P = 0.021 for light sleep time, P = 0.006 for REM sleep time).

**Table 3:** The effect of 30-day dawn-to-dusk dry fasting (DDDF) on health and fitness parameters measured by smartwatch in subjects with body mass index ≥25 kg/m^2^.

Health and Fitness Parameters	The levels measured within two weeks before 30-day DDDF		The levels measured within the fourth week of 30-day DDDF compared with the levels measured within two weeks before 30-day DDDF			The levels measured within two weeks after 30-day DDDF compared with the levels measured within two weeks before 30-day DDDF		
	Mean	SD	Mean Paired Change^a^	SD	Paired P Value	Mean Paired Change^a^	SD	Paired P Value
Average resting heart rate (bpm)^b^	66	8	−2	3	0.023	−2	3	0.072
Average heart rate (bmp)^b^	70	10	1	6	0.495	−4	6	0.051
Resting heart rate (bpm)	63	9	−1	3	0.257	−1	4	0.482
High heart rate (bpm)	127	15	−1	15	0.780	−4	12	0.284
Battery charged^c^	51	26	9	20	0.157	8	20	0.210
Battery high^c^	62	34	11	27	0.163	15	28	0.075
Battery low^c^	18	14	6	13	0.131	4	15	0.313
Battery drained^c^	−47	25	−10	23	0.123	−10	23	0.124
Intensity per week (min)^d^	223	191	−48	269	0.532	−25	101	0.389
Moderate today (min)^d^	43	29	−11	26	0.255	−13	30	0.174
Vigorous today (min)^d^	33	22	−3	4	0.326	−13	19	0.113
Stress level^e^	37	15	−6	13	0.115	−7	15	0.112
Stress rest (min)^e^	438	231	87	218	0.176	107	231	0.122
Stress low (min)^e^	184	46	−13	40	0.249	−2	61	0.897
Stress medium (min)^e^	200	106	−52	90	0.061	−59	107	0.073
Stress high (min)^e^	116	118	−48	117	0.167	−50	120	0.162
Total steps	8695	2472	−71	2809	0.929	156	2329	0.813
Distance (km)	6	2	−0.2	2	0.695	−0.03	2	0.952
Steps daily average^f^	7299	2481	−1002	3982	0.382	−1037	3020	0.240
Floors climbed up	4	4	0.2	4	0.881	1	4	0.628
Floors climbed down	4	4	−0.001	5	0.999	1	5	0.658
Calories total^g^	3409	843	−143	424	0.248	−70	455	0.590
Calories resting^g^	2214	316	−3	8	0.168	0	0	N/A
Calories active^g^	1195	628	−140	424	0.258	−70	455	0.590
Total sleep time (min)	424	77	−90	104	0.008	−4	90	0.885
Deep sleep (min)	59	43	−8	31	0.348	−3	31	0.749
Light sleep (min)	277	65	−65	89	0.021	4	70	0.854
REM (min)	102	49	−25	26	0.006	−9	28	0.304
Awake time during sleep (min)	16	12	3	24	0.666	−3	13	0.446
Average SPO_2_	92	2	−0.1	1	0.854	0.3	2	0.533
Lowest SPO_2_	84	3	−0.4	3	0.722	−0.4	4	0.731

SD = standard deviation; bmp = beat per minute; REM = Rapid eye movement; SPO2=Saturation of peripheral blood oxygen.

a Positive and negative mean paired changes indicate an increase and a decrease, respectively, in the levels measured within the fourth week of 30-day DDDF and within two weeks after 30-day DDDF compared with the levels measured within two weeks before 30-day DDDF.

b Average resting heart rate: Daily average resting heart rate during the last seven days; (one or more days were recorded in a week) [44].

c Battery charged indicates the body's available reserve energy. Low reserve energy: 0–25. Medium reserve energy: 26–50. High reserve energy: 51–75. Very high reserve energy: 76–100. A higher number indicates that the battery is charged up for the activity, whereas a lower number indicates that the body battery is drained and a break is needed (e.g., resting and having a good sleep charge the body battery; having vigorous activity, stress and poor sleep drain the body battery) [44]." and link the ref [44].

d Intensity per week: Time spent performing moderate and vigorous activity per week. Moderate today: Daily time spent performing moderate activity. Vigorous today: Daily time spent performing vigorous activity [49].

e Stress level: It is estimated from heart rate and heart rate variability data using the Firstbeat Analytics engine [45]. Stress rest: 0–25. Stress low: 26–50. Stress medium: 51–75. Stress high: 76–100 [44].

f Steps daily average: Seven-day average of step count (one or more days were recorded in a week) [49].

g Calories total: Total calories burned. Calories resting: Calories burned during inactivity. Calories active: Calories burned during physical activity [49].

### 3.4 Associations between changes in anthropometric parameters, blood pressure and heart rate and changes in circulating inflammatory cytokines/inflammatory biomarkers

The only cytokine that showed a significant correlation between its changes and changes in BMI within the fourth week of 30-day DDDF was IFN-gamma (r = −0.57, P = 0.043). There was a significant correlation between changes in waist circumference and changes in IL-15 (r = 0.70, P = 0.011) and MDC (r = 0.78, P = 0.003), between changes in hip circumference and changes in IL-1 beta (r = 0.64, P = 0.047) and MDC (r = −0.66, P = 0.040), between changes in waist to hip ratio (WHR) and changes in MDC (r = 0.90, P = 0.0003), between changes in systolic blood pressure and changes in IL-6 (r = 0.59, P = 0.034) and IP-10 (r = 0.67, P = 0.013), between changes in heart rate and changes in IL-8 (r = 0.69, P = 0.009) levels within the fourth week of 30-day DDDF compared with baseline levels.

There was also a significant correlation between changes in waist circumference and changes in MDC (r = 0.64, P = 0.018), PDGF-AB/BB (r = 0.68, P = 0.010), sCD40L (r = 0.59, P = 0.034) and VEGF-A (r = 0.68, P = 0.011), between changes in WHR and changes in IL-12p40 (r = −0.68, P = 0.015) and VGEF-A (r = 0.60, P = 0.041), between changes in diastolic blood pressure and changes in IL-12p40 (r = 0.56, P = 0.046) and CRP (r = 0.65, P = 0.017), between changes in mean arterial blood pressure and changes in CRP (r = 0.73, P = 0.005) levels within two weeks after 30-day DDDF compared with baseline levels.

No significant correlation was found between changes in weight and changes in cytokine/inflammatory biomarker levels within the fourth week of 30-day DDDF and within two weeks after 30-day DDDF compared with the levels measured within two weeks before 30-day DDDF.

### 3.5 Associations between changes in average resting heart rate and sleep parameters measured by smartwatch and changes in circulating inflammatory cytokines/inflammatory biomarkers

A significant positive correlation was observed between changes in average resting heart rate and changes in IL-8 (r = 0.57, P = 0.042) levels within the fourth week of 30-day DDDF compared with baseline levels.

There was a significant negative correlation between changes in average resting heart rate and changes in IFN-gamma (r = −0.60, P = 0.031), between changes in total sleep time and changes in GCSF (r = −0.58, P = 0.039), IL-15 (r = −0.70, P = 0.008), PDGF-AB/BB (r = −0.62, P = 0.023), and VEGF-A (r = −0.59, P = 0.034), between changes in light sleep time and changes in IL-15 (r = −0.76, P = 0.003) and IL-6 (r = −0.59, P = 0.032), between changes in awake time during sleep and changes in FLT3L (r = −0.80, P = 0.003) and IL-13 (R = −0.67, P = 0.025) levels within two weeks after 30-day DDDF compared with baseline levels. A significant positive correlation between changes in awake time during sleep and changes in IFN-gamma (r = 0.80, P = 0.003) level was also observed within two weeks after 30-day DDDF compared with baseline levels.

## 4 Discussion

Obesity is a state of chronic low-level inflammation linked to multiple autoimmune and chronic metabolic diseases and cancers [52]. This study demonstrates that DDDF regimen significantly decreased several circulating inflammatory cytokine levels, decreasing inflammation in subjects with increased BMI. There was a decrease in the mean levels of circulating cytokines within the fourth week of 30-day DDDF and within two weeks after 30-day DDDF; however, this decrease was not correlated with the decrease in body weight. We did not observe any significant change in the active calories (calories burned during physical activity) within the fourth week of the 30-day DDDF, or within two weeks after the 30-day DDDF compared with active calories at baseline. These findings suggest that the effect of DDDF on decreasing cytokine/inflammatory biomarker levels could be independent of the decrease in body weight and calories expended. Although there was no significant correlation between changes in weight and changes in circulating inflammatory cytokines, there was a significant positive correlation between changes in waist circumference and changes in specific inflammatory cytokine levels (e.g., IL-15, MDC, PDGF-AB/BB, sCD40L, and VEGF-A) within the fourth week of 30-day DDDF and/or two weeks after 30-day DDDF suggesting a potential mechanistic link between the decrease in inflammation and reduction in visceral fat.

We observed a persistent decrease in the mean levels of TNF-alpha within the fourth week of 30-day DDDF and within two weeks after the completion of 30-day DDDF (P = 0.046) (Table 2). TNF-alpha, which is a member of TNF superfamily, is a proinflammatory cytokine that plays a critical role in the development of several chronic autoimmune and inflammatory diseases (e.g., insulin resistance, type 2 diabetes mellitus, rheumatoid arthritis, inflammatory bowel disease, Alzheimer's dementia) and cancer [53,54]. Our findings align with previous studies showing decreased TNF-alpha levels after DDDF [55,56].

The subjects experienced a statistically significant decrease in the mean IL-1 beta levels within the fourth week of 30-day DDDF (P = 0.045), which persisted even within two weeks after 30-day DDDF (P = 0.024) (Table 2). The secretion of IL-1 beta follows the activation of inflammasome rather than trafficking through the endoplasmic reticulum and Golgi apparatus [57]. Inflammasome is a specialized protein complex within a cell that is activated via stress signals, triggering an inflammatory response, cytokine release, and sometimes resulting in pyroptosis which is inflammatory cell death [58]. Besides its role in inflammation, several studies showed the tumor promotor effect of IL-1 beta in various cancers, including stomach, breast, colon, pancreas, and liver [[59], [60], [61], [62], [63]]. The results of our study showing a significant decrease in the mean IL-1 beta levels within the fourth week of 30-day DDDF and within two weeks after 30-day DDDF suggest that DDDF can be considered an adjunct treatment in several cancers, and further studies are needed.

A significant decrease in the mean levels of several other cytokines was observed including IL-15 (P = 0.014), IL-1ra (P = 0.041), MDC (P = 0.013), and MIG/CXCL9 (P = 0.027) within the fourth week of 30-day DDDF, and IL-12p40 (P = 0.038) and IL-22 (P = 0.025) within two weeks after 30-day DDDF compared with the baseline levels of these cytokines (Table 2). Increased levels of these inflammatory cytokines were reported to be involved in the low-grade chronic inflammation of obesity and its complications [[64], [65], [66], [67], [68], [69]]. IL-15 is a proinflammatory cytokine implicated in the development of insulin resistance and hepatic steatosis [[64], [65]]. IL-15 knockout mice were shown to be protected from diet-induced obesity, fat accumulation in adipocytes, insulin resistance, increase in free fatty acids, and hepatic steatosis [64,65]. Likewise, IL-1ra levels were shown to be elevated in subjects with morbid obesity and improved after bariatric surgery [66]. MDC, also known as CCL22, was upregulated in obesity and linked to endothelial dysfunction [67,68]. A study showed that, in contrast to mice fed by regular chow diet, mice fed by methionine/choline-deficient diet had metabolic dysfunction-associated steatohepatitis, and significantly increased MIG/CXCL9 levels that contributed to an imbalance in the regulatory T cell to T helper 17 ratio by suppressing regulatory T cell and increasing T helper 17 cell proliferation [69]. The same study also showed that subjects with MASLD has significantly higher levels of MIG/CXCL9 levels compared with the levels in healthy control subjects [69]. The ramification of these important findings requires further inquiry.

A persistent decrease in the mean FGF-2 levels was observed within two weeks since the completion of 30-day DDDF (Table 2). These findings are congruent with the studies that showed a mechanistic link between elevated FGF-2 levels and visceral adiposity, inflammation, and tumorigenesis and between decreased FGF-2 levels and protection against visceral adiposity, inflammation and hepatic steatosis [[70], [71], [72]].

Although we observed a decrease in the mean IL-6 levels within the fourth week of the 30-day DDDF and within two weeks after the 30-day DDDF compared with baseline levels, this was not statistically significant. IL-6 and other circulating cytokine levels that we measured in the systemic venous circulation might be much higher in the portal circulation that drains visceral adipose tissue [73]. According to the portal hypothesis, the inflammatory cytokines released from visceral adipose tissue that drains to the portal circulation play a key role in the development of insulin resistance and metabolic dysfunction in obesity [[73], [74], [75], [76]]. In fact, a study conducted on 25 subjects with obesity undergoing gastric bypass surgery showed that IL-6 concentrations in the portal vein were much higher than the IL-6 concentrations in the radial artery [73]. Additionally, there was a positive correlation between portal vein IL-6 and radial artery systemic CRP concentrations, suggesting a mechanistic connection between the inflammation in visceral adipose tissue and systemic chronic low-grade inflammation [73]. Despite this potential mechanistic connection between visceral adipose tissue and systemic inflammation, inflammatory cytokines released from visceral adipose tissue rather than systemic inflammation alone appear to play a critical role in the development of insulin resistance [74]. A murine study showed that in contrast to fat transplanted to the parietal peritoneum that drains to the systemic circulation, only fat transplanted to the mesentery that drains to the portal vein resulted in hepatic insulin resistance [74]. The results are evidence of the portal hypothesis and show a direct association between inflammatory cytokines released from visceral adipose tissue that drains to the portal vein and the development of insulin resistance [74]. We not only observed a significant decrease in multiple circulating inflammatory cytokines, but we also noted a significant positive correlation between changes in specific inflammatory cytokines (e.g., IL-15, MDC PDGF-AB/BB, sCD40L and VEGF-A) and changes in the levels of surrogate markers of visceral adiposity within the fourth week of 30-day DDDF and/or two weeks after 30-day DDDF. Considering the results of these prior studies and our study results, DDDF should be considered a potent treatment to reduce chronic inflammation in visceral obesity, and further studies are needed.

Regarding health and fitness parameters, subjects experienced a significant decrease in the mean average resting heart rate measured by smartwatch (P = 0.023) within the fourth week of 30-day DDDF (Table 3). There was also a positive correlation between changes in average resting heart rate and changes in IL-8 (r = 0.57, P = 0.042) levels within the fourth week of 30-day DDDF compared with baseline level. A similar positive correlation between resting heart rate and inflammation was previously reported [77]. The multi-ethnic study of atherosclerosis (MESA) showed that increased resting heart rate was an independent risk factor for higher levels of inflammation [77]. The multivariate model adjusted for physical activity and atrioventricular nodal blocker treatment showed a significant association between the increase in resting heart rate and increase in inflammatory biomarkers, including high-sensitivity CRP, IL-6, and fibrinogen [77].

The study subjects also had a significant shortening in total sleep, light sleep, and REM sleep times within the fourth week of 30-day DDDF (Table 3). These findings appear to be related to the changes in their sleep/wake and mealtime schedules, nocturnal activities, and nighttime light exposure during 30-day DDDF [78]. Analyses that controlled for environmental factors showed that DDDF was not associated with changes in the cyclical pattern of normal sleep [78]. We did not observe any significant correlation between changes in total sleep, deep sleep, light sleep, and REM sleep times and changes in circulating cytokine levels within the fourth week of 30-day DDDF compared with baseline level.

The primary strength of our study is the simultaneous measurement of a total of 21 cytokines/inflammatory biomarkers both during and after 30-DDDF. Assessing the levels of these cytokines showed that even after ending 30-day DDDF, the levels of several cytokines significantly decreased.

We used a smartwatch to estimate the health and fitness parameters. Because many parameters tracked by the smartwatch are estimated instead of measured directly, the results should be interpreted cautiously, particularly the parameters related to sleep cycles and calories. As this is a religious fast with predetermined dates, all subjects had to start and end the 30-day DDDF on the same day. Therefore, scheduling all 13 subjects’ visits on the same day before, during, and after the study was challenging. This scheduling challenge might have caused some variance in our data. Similar to our study, other religious fasting studies also reported flexible dates for study visits [17,79].

## 5 Conclusions

DDDF with meals immediately before and after fasting period is a potent form of intermittent fasting compliant with the human circadian rhythm, reinforcing the phase of all peripheral clocks to be aligned with the phase of the central clock at dawn and dusk (critical periods for human metabolism) [19,43,44]. The relatively short duration of the dry fasting period of DDDF (from dawn to dusk), specifically in winter compared to prolonged wet or dry fasting regimens (e.g., 24-h or longer fasting), prevents starvation, starvation-induced refeeding syndrome [45], and dehydration. In contrast to water/wet fasting, dry fasting prevents protein breakdown as an alternative energy source during fasting and, therefore, protects against skeletal muscle and vital body weight loss that can occur with water/wet fasting [30]. DDDF appears to be a unique treatment to reduce low-grade chronic systemic inflammation caused by visceral obesity, without compromising the body's innate and adaptive immune mechanisms. An alternative treatment option to suppress inflammation and cytokine release would be pharmaceutical blockage, which could significantly impair immune function and develop opportunistic infections and multiple malignancies.

## Funding

Research reported in this publication was supported in part indirectly by National Institute of Diabetes and Digestive and Kidney Diseases of the National Institutes of Health under award number P30DK056338, which funds the Study Design and Clinical Research Core of the Texas Medical Center Digestive Diseases Center, and National Institute on Aging of the National Institutes of Health under award number U19AG065169. Research reported in this publication was also supported in part by Dora Roberts Foundation and an unrestricted grant from D.R. and G.P. Laws. The content is solely the responsibility of the authors and does not necessarily represent the official views of the National Institutes of Health.

## CRediT authorship contribution statement

**Zahraa Al lami:** Data curation, Investigation, Project administration, Validation, Writing – review & editing. **Miray Kurtca:** Data curation, Investigation, Project administration, Validation, Writing – review & editing. **Moin Uddin Atique:** Investigation, Project administration, Writing – review & editing, Data curation, Formal analysis, Validation. **Antone R. Opekun:** Data curation, Funding acquisition, Investigation, Project administration, Resources, Supervision, Validation, Writing – review & editing, Methodology. **Mohamad S. Siam:** Project administration, Writing – review & editing, Validation. **Prasun K. Jalal:** Resources, Writing – review & editing, Funding acquisition. **Bijan Najafi:** Investigation, Project administration, Resources, Supervision, Validation, Writing – review & editing. **Sridevi Devaraj:** Data curation, Investigation, Methodology, Project administration, Resources, Supervision, Validation, Writing – review & editing. **Ayse L. Mindikoglu:** Conceptualization, Data curation, Formal analysis, Funding acquisition, Investigation, Methodology, Project administration, Resources, Software, Supervision, Validation, Writing – original draft, Writing – review & editing.

## Declaration of competing interest

None of the authors has a conflict of interest.

## References

[1] Wolfenden L., Ezzati M., Larijani B., Dietz W. (2019). The challenge for global health systems in preventing and managing obesity. Obes Rev.

[2] Blüher M. (2019). Obesity: global epidemiology and pathogenesis. Nat Rev Endocrinol.

[3] Boutari C., Mantzoros C.S., A (2022). 2022 update on the epidemiology of obesity and a call to action: as its twin COVID-19 pandemic appears to be receding, the obesity and dysmetabolism pandemic continues to rage on. Metabolism.

[4] Khandekar M.J., Cohen P., Spiegelman B.M. (2011). Molecular mechanisms of cancer development in obesity. Nat Rev Cancer.

[5] Salvestrini V., Sell C., Lorenzini A. (2019). Obesity may accelerate the aging process. Front Endocrinol.

[6] Mitchell N.S., Catenacci V.A., Wyatt H.R., Hill J.O. (2011). Obesity: overview of an epidemic. Psychiatr Clin North Am.

[7] Obesity and overweight. Available at https://www.who.int/news-room/fact-sheets/detail/obesity-and-overweight. Accessed on September 28, 2023.

[8] Schwartz M.W., Seeley R.J., Zeltser L.M., Drewnowski A., Ravussin E., Redman L.M., Leibel R.L. (2017). Obesity pathogenesis: an endocrine society scientific statement. Endocr Rev.

[9] Romieu I., Dossus L., Barquera S., Blottière H.M., Franks P.W., Gunter M., Hwalla N., Hursting S.D., Leitzmann M., Margetts B., Nishida C., Potischman N., Seidell J., Stepien M., Wang Y., Westerterp K., Winichagoon P., Wiseman M., Willett W.C. (2017). Energy balance and obesity: what are the main drivers?. Cancer Causes Control.

[10] Schroeder B.O., Bäckhed F. (2016). Signals from the gut microbiota to distant organs in physiology and disease. Nat Med.

[11] Hall K.D., Guo J. (2017). Obesity energetics: body weight regulation and the effects of diet composition. Gastroenterology.

[12] Haczeyni F., Bell-Anderson K.S., Farrell G.C. (2018). Causes and mechanisms of adipocyte enlargement and adipose expansion. Obes Rev.

[13] Hildebrandt X., Ibrahim M., Peltzer N. (2023). Cell death and inflammation during obesity: “Know my methods, WAT(son)”. Cell Death Differ.

[14] Hotamisligil G.S., Shargill N.S., Spiegelman B.M. (1993). Adipose expression of tumor necrosis factor-α: direct role in obesity-linked insulin resistance. Science.

[15] Kawai T., Autieri M.V., Scalia R. (2021). Adipose tissue inflammation and metabolic dysfunction in obesity. American Journal of Physiology-Cell Physiology.

[16] Faris M.e.A.-I.E., Jahrami H.A., Obaideen A.A., Madkour M.I. (2019). Impact of diurnal intermittent fasting during Ramadan on inflammatory and oxidative stress markers in healthy people: systematic review and meta-analysis. Journal of Nutrition & Intermediary Metabolism.

[17] Faris M.A., Kacimi S., Al-Kurd R.A., Fararjeh M.A., Bustanji Y.K., Mohammad M.K., Salem M.L. (2012). Intermittent fasting during Ramadan attenuates proinflammatory cytokines and immune cells in healthy subjects. Nutr Res.

[18] Abdulsada M.M., Wilhelm Z.R., Opekun A.R., Devaraj S., Jalal P.K., Mindikoglu A.L. (2021). The effect of four-week intermittent fasting from dawn to sunset on circulating brain-derived neurotrophic factor levels in subjects with metabolic syndrome and healthy subjects. Metabolism Open.

[19] Mindikoglu A.L., Park J., Opekun A.R., Abdulsada M.M., Wilhelm Z.R., Jalal P.K., Devaraj S., Jung S.Y. (2022). Dawn-to-dusk dry fasting induces anti-atherosclerotic, anti-inflammatory, and anti-tumorigenic proteome in peripheral blood mononuclear cells in subjects with metabolic syndrome. Metabol Open.

[20] Zouhal H., Bagheri R., Ashtary-Larky D., Wong A., Triki R., Hackney A.C., Laher I., Abderrahman A.B. (2020). Effects of Ramadan intermittent fasting on inflammatory and biochemical biomarkers in males with obesity. Physiol Behav.

[21] Faris M.A.E., Madkour M.I., Obaideen A.K., Dalah E.Z., Hasan H.A., Radwan H., Jahrami H.A., Hamdy O., Mohammad M.G. (2019). Effect of Ramadan diurnal fasting on visceral adiposity and serum adipokines in overweight and obese individuals. Diabetes Res Clin Pract.

[22] Wehr T.A., Aeschbach D., Duncan W.C. (2001). Evidence for a biological dawn and dusk in the human circadian timing system. J Physiol.

[23] Wehr T.A., Schwartz P.J., Turner E.H., Feldman-Naim S., Drake C.L., Rosenthal N.E. (1995). Bimodal patterns of human melatonin secretion consistent with a two-oscillator model of regulation. Neurosci Lett.

[24] Witbracht M., Keim N.L., Forester S., Widaman A., Laugero K. (2015). Female breakfast skippers display a disrupted cortisol rhythm and elevated blood pressure. Physiology & behavior.

[25] Nas A., Mirza N., Hagele F., Kahlhofer J., Keller J., Rising R. (2017). Impact of breakfast skipping compared with dinner skipping on regulation of energy balance and metabolic risk. Am J Clin Nutr.

[26] Smith K.J., Gall S.L., McNaughton S.A., Blizzard L., Dwyer T., Venn A.J. (2010). Skipping breakfast: longitudinal associations with cardiometabolic risk factors in the Childhood Determinants of Adult Health Study. Am J Clin Nutr.

[27] Qin L.Q., Li J., Wang Y., Wang J., Xu J.Y., Kaneko T. (2003). The effects of nocturnal life on endocrine circadian patterns in healthy adults. Life Sci.

[28] Gu C., Brereton N., Schweitzer A., Cotter M., Duan D., Børsheim E., Wolfe R.R., Pham L.V., Polotsky V.Y., Jun J.C. (2020). Metabolic effects of late dinner in healthy volunteers-A randomized crossover clinical trial. J Clin Endocrinol Metab.

[29] Lowe D.A., Wu N., Rohdin-Bibby L., Moore A.H., Kelly N., Liu Y.E. (2020). Effects of time-restricted eating on weight loss and other metabolic parameters in women and men with overweight and obesity: the TREAT randomized clinical trial. JAMA Intern Med.

[30] Dunning A. (2020).

[31] Geelen G., Greenleaf J.E., Keil L.C. (1996). Drinking-induced plasma vasopressin and norepinephrine changes in dehydrated humans. J Clin Endocrinol Metab.

[32] Graça F.A., Gonçalves D.A., Silveira W.A., Lira E.C., Chaves V.E., Zanon N.M. (2013). Epinephrine depletion exacerbates the fasting-induced protein breakdown in fast-twitch skeletal muscles. Am J Physiol Endocrinol Metab.

[33] Labrie F., Giguere V., Proulx L., Lefevre G. (1984). Interactions between CRF, epinephrine, vasopressin and glucocorticoids in the control of ACTH secretion. J Steroid Biochem.

[34] Valenta L.J., Elias A.N., Eisenberg H. (1986). ACTH stimulation of adrenal epinephrine and norepinephrine release. Horm Res.

[35] Pedersen S.B., Bak J.F., Holck P., Schmitz O., Richelsen B. (1999). Epinephrine stimulates human muscle lipoprotein lipase activity in vivo. Metabolism.

[36] Palazzo A.J., Malik K.U., Weis M.T. (1991). Vasopressin stimulates the mobilization and metabolism of triacylglycerol in perfused rabbit hearts. Am J Physiol.

[37] Abdalla A.H., Shaheen F.A., Rassoul Z., Owda A.K., Popovich W.F., Mousa D.H., al-Hawas F., al-Sulaiman M.H., al-Khader A.A. (1998). Effect of Ramadan fasting on Moslem kidney transplant recipients. Am J Nephrol.

[38] Cheah S.H., Ch'ng S.L., Husain R., Duncan M.T. (1990). Effects of fasting during Ramadan on urinary excretion in Malaysian Muslims. Br J Nutr.

[39] Leiper J.B., Molla A.M., Molla A.M. (2003). Effects on health of fluid restriction during fasting in Ramadan. Eur J Clin Nutr.

[40] Aslam M., Wilson J.V. (1989). Clinical problems during the fast of Ramadan. Lancet.

[41] Koppold-Liebscher D.A., Klatte C., Demmrich S., Schwarz J., Kandil F.I., Steckhan N., Ring R., Kessler C.S., Jeitler M., Koller B., Ananthasubramaniam B., Eisenmann C., Mähler A., Boschmann M., Kramer A., Michalsen A. (2021). Effects of daytime dry fasting on hydration, glucose metabolism and circadian phase: a prospective exploratory cohort study in bahá'í volunteers. Front Nutr.

[42] Mindikoglu A.L., Abdulsada M.M., Jain A., Choi J.M., Jalal P.K., Devaraj S. (2020). Intermittent fasting from dawn to sunset for 30 consecutive days is associated with anticancer proteomic signature and upregulates key regulatory proteins of glucose and lipid metabolism, circadian clock, DNA repair, cytoskeleton remodeling, immune system and cognitive function in healthy subjects. J Proteomics.

[43] Mindikoglu A.L., Abdulsada M.M., Jain A., Jalal P.K., Devaraj S., Wilhelm Z.R., Opekun A.R., Jung S.Y. (2020). Intermittent fasting from dawn to sunset for four consecutive weeks induces anticancer serum proteome response and improves metabolic syndrome. Sci Rep.

[44] Bhatti S.I., Mindikoglu A.L. (2022). The impact of dawn to sunset fasting on immune system and its clinical significance in COVID-19 pandemic. Metabol Open.

[45] Crook M.A., Hally V., Panteli J.V. (2001). The importance of the refeeding syndrome. Nutrition.

[46] Papagiannopoulos-Vatopaidinos I.E., Papagiannopoulou M., Sideris V. (2020). Dry fasting physiology: responses to hypovolemia and hypertonicity. Complement Med Res.

[47] Borruel S., Moltó J.F., Alpañés M., Fernández-Durán E., Álvarez-Blasco F., Luque-Ramírez M., Escobar-Morreale H.F. (2014). Surrogate markers of visceral adiposity in young adults: waist circumference and body mass index are more accurate than waist hip ratio, model of adipose distribution and visceral adiposity index. PLoS One.

[48] Fasting times. QUR'AN. Surah Al-Baqarah (2:187).

[49] Garmin Vivosmart® 4 Owner's Manual. Available at https://www8.garmin.com/manuals/webhelp/vivosmart4/EN-US/GUID-2A2C3E97-390C-4123-8261-2A86C5E11549-homepage.html. Accessed on August 17, 2023.

[50] Firstbeat Analytics (2015). https://www.firstbeatanalytics.com/en/.

[51] SAS software. http://www.Sas.Com/. The data analysis for this paper was generated using SAS software, Version 9.4 of the SAS System for Windows. Copyright © 2020 SAS Institute Inc. SAS and all other SAS Institute Inc. product or service names are registered trademarks or trademarks of SAS Institute Inc, Cary, NC, USA.

[52] Hotamisligil G.S. (2006). Inflammation and metabolic disorders. Nature.

[53] Gene (2004). Bethesda (MD): national library of medicine (US). National Center for Biotechnology Information.

[54] Aggarwal B.B. (2003). Signalling pathways of the TNF superfamily: a double-edged sword. Nat Rev Immunol.

[55] Mushtaq R., Akram A., Mushtaq R., Khwaja S., Ahmed S. (2019). The role of inflammatory markers following Ramadan Fasting. Pak J Med Sci.

[56] Unalacak M., Kara I.H., Baltaci D., Erdem O., Bucaktepe P.G. (2011). Effects of Ramadan fasting on biochemical and hematological parameters and cytokines in healthy and obese individuals. Metab Syndr Relat Disord.

[57] Lopez-Castejon G., Brough D. (2011). Understanding the mechanism of IL-1β secretion. Cytokine Growth Factor Rev.

[58] de Zoete M.R., Palm N.W., Zhu S., Flavell R.A. (2014). Inflammasomes. Cold Spring Harb Perspect Biol.

[59] Tu S., Bhagat G., Cui G., Takaishi S., Kurt-Jones E.A., Rickman B., Betz K.S., Penz-Oesterreicher M., Bjorkdahl O., Fox J.G., Wang T.C. (2008). Overexpression of interleukin-1beta induces gastric inflammation and cancer and mobilizes myeloid-derived suppressor cells in mice. Cancer Cell.

[60] Arendt L.M., McCready J., Keller P.J., Baker D.D., Naber S.P., Seewaldt V., Kuperwasser C. (2013). Obesity promotes breast cancer by CCL2-mediated macrophage recruitment and angiogenesis. Cancer Res.

[61] Maker A.V., Katabi N., Qin L.X., Klimstra D.S., Schattner M., Brennan M.F., Jarnagin W.R., Allen P.J. (2011). Cyst fluid interleukin-1beta (IL1beta) levels predict the risk of carcinoma in intraductal papillary mucinous neoplasms of the pancreas. Clin Cancer Res.

[62] Kohga K., Tatsumi T., Tsunematsu H., Aono S., Shimizu S., Kodama T. (2012). Interleukin-1β enhances the production of soluble MICA in human hepatocellular carcinoma. Cancer Immunol Immunother.

[63] Kaler P., Augenlicht L., Klampfer L. (2009). Macrophage-derived IL-1beta stimulates Wnt signaling and growth of colon cancer cells: a crosstalk interrupted by vitamin D3. Oncogene.

[64] Lacraz G., Rakotoarivelo V., Labbé S.M., Vernier M., Noll C., Mayhue M., Stankova J., Schwertani A., Grenier G., Carpentier A., Richard D., Ferbeyre G., Fradette J., Rola-Pleszczynski M., Menendez A., Langlois M.F., Ilangumaran S., Ramanathan S. (2016). Deficiency of interleukin-15 confers resistance to obesity by diminishing inflammation and enhancing the thermogenic function of adipose tissues. PLoS One.

[65] Cepero-Donates Y., Lacraz G., Ghobadi F., Rakotoarivelo V., Orkhis S., Mayhue M., Chen Y.G., Rola-Pleszczynski M., Menendez A., Ilangumaran S., Ramanathan S. (2016). Interleukin-15-mediated inflammation promotes non-alcoholic fatty liver disease. Cytokine.

[66] Meier C.A., Bobbioni E., Gabay C., Assimacopoulos-Jeannet F., Golay A., Dayer J.M. (2002). IL-1 receptor antagonist serum levels are increased in human obesity: a possible link to the resistance to leptin?. J Clin Endocrinol Metab.

[67] Gene (2004). Bethesda (MD): national library of medicine (US). National Center for Biotechnology Information.

[68] Hueso L., Marques P., Morant B., Gonzalez-Navarro H., Ortega J., Real J.T., Sanz M.J., Piqueras L. (2023). CCL17 and CCL22 chemokines are upregulated in human obesity and play a role in vascular dysfunction. Front Endocrinol.

[69] Li L., Xia Y., Ji X., Wang H., Zhang Z., Lu P., Ding Q., Wang D., Liu M. (2021). MIG/CXCL9 exacerbates the progression of metabolic-associated fatty liver disease by disrupting Treg/Th17 balance. Exp Cell Res.

[70] Chakraborty D., Benham V., Bullard B., Kearney T., Hsia H.C., Gibbon D., Demireva E.Y., Lunt S.Y., Bernard J.J. (2017). Fibroblast growth factor receptor is a mechanistic link between visceral adiposity and cancer. Oncogene.

[71] ZhuGe D.L., Javaid H.M.A., Sahar N.E., Zhao Y.Z., Huh J.Y. (2020). Fibroblast growth factor 2 exacerbates inflammation in adipocytes through NLRP3 inflammasome activation. Arch Pharm Res (Seoul).

[72] Li H., Zhang X., Huang C., Liu H., Zhang Q., Sun Q., Jia Y., Liu S., Dong M., Hou M., Liu Y., Lin H. (2021). FGF2 disruption enhances thermogenesis in brown and beige fat to protect against adiposity and hepatic steatosis. Mol Metab.

[73] Fontana L., Eagon J.C., Trujillo M.E., Scherer P.E., Klein S. (2007). Visceral fat adipokine secretion is associated with systemic inflammation in obese humans. Diabetes.

[74] Rytka J.M., Wueest S., Schoenle E.J., Konrad D. (2011). The portal theory supported by venous drainage-selective fat transplantation. Diabetes.

[75] Montague C.T., O'Rahilly S. (2000). The perils of portliness: causes and consequences of visceral adiposity. Diabetes.

[76] Després J.P., Moorjani S., Lupien P.J., Tremblay A., Nadeau A., Bouchard C. (1990). Regional distribution of body fat, plasma lipoproteins, and cardiovascular disease. Arteriosclerosis: An Official Journal of the American Heart Association, Inc..

[77] Whelton S.P., Narla V., Blaha M.J., Nasir K., Blumenthal R.S., Jenny N.S., Al-Mallah M.H., Michos E.D. (2014). Association between resting heart rate and inflammatory biomarkers (high-sensitivity C-reactive protein, interleukin-6, and fibrinogen) (from the Multi-Ethnic Study of Atherosclerosis). Am J Cardiol.

[78] Qasrawi S.O., Pandi-Perumal S.R., BaHammam A.S. (2017). The effect of intermittent fasting during Ramadan on sleep, sleepiness, cognitive function, and circadian rhythm. Sleep Breath.

[79] Nematy M., Alinezhad-Namaghi M., Rashed M.M., Mozhdehifard M., Sajjadi S.S., Akhlaghi S., Sabery M., Mohajeri S.A., Shalaey N., Moohebati M., Norouzy A. (2012). Effects of Ramadan fasting on cardiovascular risk factors: a prospective observational study. Nutr J.

